# High *T_g_*, Bio-Based Isosorbide Methacrylate Resin Systems for Vat Photopolymerization

**DOI:** 10.3390/polym15092007

**Published:** 2023-04-24

**Authors:** Xi Chu, Jianwei Tu, Heather R. Berensmann, John J. La Scala, Giuseppe R. Palmese

**Affiliations:** 1 Chemical and Biological Engineering, Drexel University, Philadelphia, PA 19104, USA; 2 Department of Chemical Engineering, Rowan University, Glassboro, NJ 08028, USA; 3 Army Research Laboratory, 4600 Deer Creek Loop, Aberdeen Proving Grounds, MD 21005, USA

**Keywords:** 3D printing, isosorbide methacrylate, biobased, vat photopolymerization, *T_g_*

## Abstract

The use of isosorbide-derived polymers has garnered significant attention in recent decades as a high-performance, renewable material sourced from biomass. Of particular interest is isosorbide methacrylate, which possesses low viscosity (<500 cps), high thermal properties (*T_g_* ≈ 220 °C), and high modulus (>4 GPa). These characteristics present a promising opportunity to replace BPA-derived methacrylate compounds in various applications. This investigation aims to synthesize and characterize isosorbide-based low-viscosity resin systems for 3D printing. The resin blends are composed of isosorbide methacrylate and two bio-renewable methacrylates, furfuryl methacrylate (FM) and bis-hydroxymethyl-furan methacrylate (BHMF-M), polymerized through a digital light processing (DLP) technique. The addition of the bio-based co-monomers serves to enhance the fracture toughness of the brittle isosorbide methacrylate crosslinked homopolymer (*G_Ic_* = 37 J/m^2^). The resulting polymers exhibit *T_g_* values greater than 200 °C and *G_Ic_* around 100 J/m^2^. These resin systems hold potential for imparting high bio-based content to polymers used in additive manufacturing for high-performance applications.

## 1. Introduction

Additive Manufacturing (AM), commonly referred to as 3D printing, is poised to play a significant role in the upcoming industrial revolution, as researchers are exploring ways to optimize and improve the production of many components [1,2,3]. AM holds the potential for faster production of complex parts for jet engines, hearing aids, and gas turbines compared to traditional molding methods [4,5,6,7,8,9,10,11,12]. The uses of AM extend to dental and medical applications, as well as consumer goods [4,5,6,7,8,9,10,11,12,13]. In the AM printing process, 2D layers are combined sequentially to form a 3D structure, enabling the creation of complex geometries with efficient use of time and reduced waste. A wide range of materials, including organic polymers, ceramics, and metal alloys, can be processed using various printing technologies such as vat polymerization, material extrusion, and powder bed fusion, among others [1,2,3]. One of the most widely used vat polymerization printing techniques is digital light processing (DLP), which adopts UV-irradiation to cure photo-sensitive polymers and pre-polymers on a curing platform in a layer-by-layer fashion [2,3,7,11,13,14,15].

Petroleum-derived (meth)acrylate resin systems, particularly those sourced from bisphenol-A (BPA), have been widely studied and used in 3D printing for their rapid curing, excellent adhesion, and acceptable thermal and mechanical properties [16,17,18]. However, these BPA-based (meth)acrylate resins are non-renewable and have a high viscosity, limiting their processability during printing [18,19].

**Figure 1:**
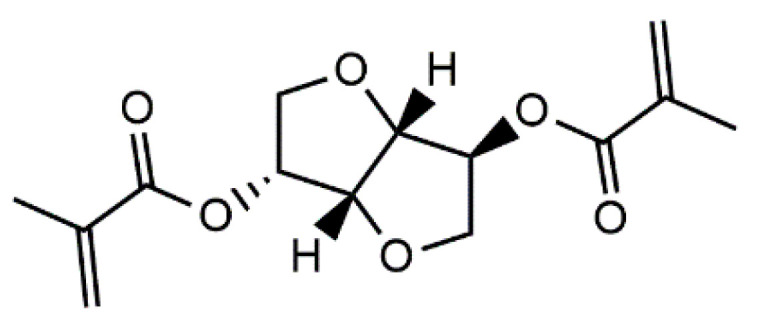
Chemical structure of isosorbide methacrylate.

Isosorbide methacrylate (IM)—in this case, the dimethacrylate of isosorbide—was introduced as a bio-based, low-viscosity resin for high-performance thermosetting applications in 2013 by Sadler et al. [20]. The chemical structure of isosorbide methacrylate is given in Figure 1. IM monomers can undergo free-radical reaction to form a thermosetting polymer with high extent of cure (85%) and a *T_g_* greater than 220 °C. The polymer also exhibits good thermal stability and a high modulus [20]. These properties make IM potentially suitable for incorporation into AM resin formulations.

Lastovickova et al. developed a series of photocurable IM-based resin systems for use in additive manufacturing. These resin systems have low viscosity (<140 cP at 25 °C), high *T_g_* (159–231 °C), and moderate glassy state modulus (2–2.8 GPa) [21]. However, IM-based resin systems exhibit brittleness, as revealed by the critical strain energy release rate *G_Ic_* value of 26 J/m^2^ (brittle for structural applications). This brittleness is attributed to the high crosslinking density and the rigid bicyclic core structure of the IM monomer.

A common method for toughening vinyl ester resins is the incorporation of diluents such as styrene as chain extenders via copolymerization. This approach reduces viscosity and delays gelation during manufacturing processes while still providing high *T_g_* polymers (>100 °C) [17,22,23]. However, the high volatility of co-monomers such as styrene poses environmental and health concerns, driving the search for bio-mass alternatives in both industry and academic fields [24,25,26,27].

**Figure 2:**
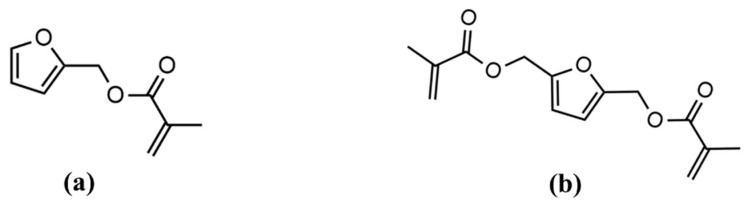
Chemical structures of (**a**) Furfuryl methacrylate (FM) and (**b**) bis-hydroxymethyl-furan methacrylate (BHMF-M).

Furan building blocks derived from cellulose and hemicellulose have garnered significant attention as a bio-renewable alternative to petroleum-based aromatics due to their high modulus, comparable *T_g_*, and excellent flame retardancy properties [28,29,30,31,32]. One of the well-studied furan-derived chemicals is 2,5-bis-(hydroxy-methyl) furan (BHMF). The esterification of BHMF to synthesize 2,5-bis(hydroxymethyl)furan methacrylate (BHMF-M) was reported by Hoydonckx et al. in 2007 [33]. The chemical structure of BHMF-M is given Figure 2. Subsequently, Hong et al. in 2019 reported a N-heterocyclic carbene (NHC)-catalyzed proton-transfer polymerization (HTP) pathway to convert BHMF-M into unsaturated polyesters. The resulting BHMF-M polyester showed improved char yield (~10 wt% residue as stable carbonaceous materials at 650 °C) compared to non-renewable systems. Furthermore, BHMF-M polyesters underwent Diels–Alder reactions to produce more robust polyester materials with the highest degree of crosslinking and char yield (31.4 wt% at 650 °C) among the systems studied [28]. Our research group has evaluated the properties of BHMF-M resin when cured with 20 wt% styrene using dynamic mechanical analysis. This BHMF-M-Styrene blend produced a polymer with high *T_g_* (150 °C), high storage modulus at room temperature (*E’* = 5.2 GPa), and low viscosity, as summarized in Table 1. Previous studies have also suggested that furan containing monomers have the potential to impart high toughness [29,32]. This phenomenon can be attributed to physical interactions associated with furan rings, resulting in the high packing efficiency of the polymer. Therefore, BHMF-M is a potential candidate as a low-viscosity toughening comonomer for bio-renewable printable resin formulations in additive manufacturing applications.

The objective of this investigation is to enhance the toughness of isosorbide methacrylate (IM) polymers through co-polymerization while maintaining high glass transition temperature and processability. The BHMF-M was copolymerized with IM via photo-polymerization at weight fractions ranging from 20% to 80%. The thermomechanical and mechanical properties of the resulting polymers were extensively studied. In addition, furfuryl methacrylate (FM), another plant-based methacrylate, was utilized as a mono-methacrylate chain extender to produce 3D-printed polymers with IM at the same weight fractions. Both low-viscosity bio-derived compounds have the potential to serve as alternatives to styrene as diluents, offering high *T_g_*, high modulus, and improved toughness. The chemical structures of FM and BHMF-M are shown in Figure 2, and Table 1 provides the viscosity of monomers, as well as storage modulus, loss modulus *T_g_*, and tan *δ*, *T_g_*, of the free-radically cured polymers of IM, FM, and BHMF-M/Styrene. 

## 2. Experimental

### 2.1. Materials

The 2,5-Bis(hydroxy-methyl) furan (BHMF) (98.8%) was purchased from Pennakem (Memphis, TN, USA). Dianhydro-D-glucitol (98%), methacrylic anhydride (94% with 2000 ppm topanol A as inhibitor), 4-dimethylaminopyridine (DMAP, 99%), sodium chloride (99%), hydroquinone (99%), sodium bicarbonate (99%), furfuryl methacrylate (FM, 97% with 200 ppm monomethyl ether hydroquinone as inhibitor) and phenylbis (2,4,6-trimethylbenzoyl) phosphine oxide (PPO, 97%) were purchased from Sigma-Aldrich (St. Louis, MO, USA). Products were used without further purification.

### 2.2. Synthesis and Chracterization of Bio-Derived Methacrylates

**Figure 3:**
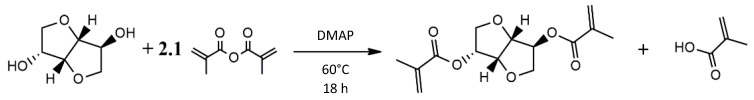
Reaction scheme for the preparation of Isosorbide methacrylate (IM) [20].

Synthesis of Isosorbide Methacrylate: IM was prepared by the reaction shown in Figure 3 [20]. Isosorbide (120 g, 0.82 mol) was combined with DMAP (5.83 g, 47.8 mmol) in a 1000 mL three-necked round bottom flask (RBF). The compounds in the RBF were placed in a 60 °C oil bath equipped with a magnetic stir bar. After about two hours, the contents of the flask became a clear and transparent liquid, free of solids. Subsequently, methacrylic anhydride (269 g, 1.75 mol) was added dropwise in slight stoichiometric excess using a pressure equalizing addition funnel and left to react with the isosorbide for 18 h at 60 °C. The product was collected and washed with 350 g of saturated aqueous sodium bicarbonate solution four times. The organic phase was then washed with deionized water and brine, leaving a colorless clear liquid resin [^1^H NMR (CDCl_3_ *δ* = 7.27) − IM, *δ*: 6.16 (s, 1H), 6.10 (s, 1H), 5.61 (s, 1H), 5.59 (s, 1H), 5.25 (s, 1H), 5.20 (q, 1H), 4.90 (t, 1H), 4.53 (d, 1H), 3.99 (s, 2H), 3.96 (d of d, 1H), 3.89 (d of d, 1H), 1.96 (t, 3H), 1.92 (t, 3H)]. The purity of IM was found to be 97% by taking the ratio of three times the peak area at *δ* = 4.53, corresponding to one of the isosorbide bridge hydrogens, to the peak at *δ* = 1.96 corresponding to the three hydrogens on one of the methyl groups of the methacrylate. The yield following the washing steps was 85–92% based on isosorbide over several synthesis runs.

**Figure 4:**
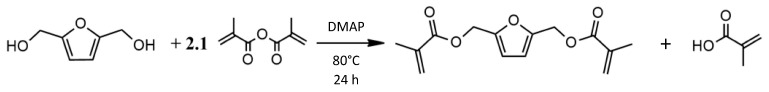
Reaction scheme for the preparation of 2,5-bis(hydroxy-methyl) furan methacrylate (BHMF-M).

Synthesis of 2,5-Bis(hydroxy-methyl) Furan Methacrylate: BHMF-M was prepared based on the reaction scheme shown in Figure 4, using the following procedure. A 1000 mL three-necked RBF equipped with a pressure equalizing addition funnel, a thermometer, a condenser, and a magnetic stirring bar inside was used. BHMF (80 g) DMAP (7.63 g) were charged into the flask. The flask was kept at 80 °C using an oil bath to melt 2,5-Bis(hydroxy-methyl) furan (BHMF) and DMAP. Methacrylic anhydride (202.13 g) was added dropwise for a period of 1 h. The reaction was carried out at 80 °C for 24 h with continuous stirring. The product was collected and washed with 300 mL saturated sodium bicarbonate aqueous solution four times. The organic phase was then washed with deionized water and brine, leaving a brown liquid [^1^H NMR (CDCl_3_ *δ* = 7.27) BHMF-M, *δ*: 6.40 (s, 2H), 6.14 (s, 2H), 5.59 (t, 2H), 5.12 (s, 4H), 1.96 (t, 6H)]. By taking the ratio of three times the peak area at *δ* = 6.40 corresponding to the two hydrogens on the furan ring to the that of the peak at *δ* = 1.96 corresponding to the six hydrogens on the methyl groups of the methacrylate groups, the purity of BHMF-M was found to be 98%. The yield following the washing steps was 85% relative to BHMF.

^1^H-NMR Spectroscopy: ^1^H-NMR measurements were conducted with a Varian VXR-Unity 500 (500 Hz) instrument (Cranford, NJ, USA) with spectral window of ±2000 Hz, 32 scans at 293 K and 90° pulse width to confirm the structure of synthesized BHMF-M and IM as given above. 

Acid Titration: Acid titration was carried out following ASTM D974–14e2 [34] standard to determine the amount of residual methacrylic acid from the synthesis and washing process. Acid number of various batches of resin were all titrated to be below 3 mg KOH/g. 

### 2.3. Preparation and Characterization of IM-Based Resin Blends and Photocured Polymers

Preparation of IM-based Resin Blends: IM was blended with FM and BHMF-M separately as co-monomers at weight fractions ranging from 20% to 80% and 0.7 PPO as the photo-initiator using a THINKY centrifugal mixer (Laguna Hills, CA, USA) at a speed of 1600 rpm for 5 min followed by a de-gassing step for 1 min. 

Viscosity: The viscosity of BHMF-M/IM and FM/IM resin blends was measured using an AR2000 ex rheometer at 21 °C (TA Instruments, New Castle, DE, USA) with 40 mm flat plate configuration using a shear rate range from 0.01 to 1000 s^−1^, with 10 measurements recorded per decade. Shear stress was measured every 2 s at each shear rate. Viscosity was reported as the average of three measurements at a shear rate of 1000 s^−1^. 

**Figure 5:**
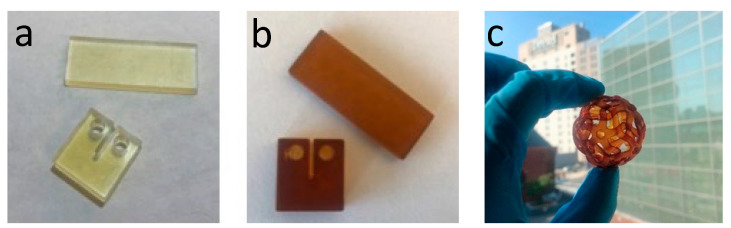
DMA and fracture toughness samples printed using FM/IM resins (**a**), BHMF-M/IM resins (**b**), and a complex open structure printed using a 20% BHMF-M/80% IM resin blend (**c**).

Photo-Curing and Post-Curing: Compact tension and dynamic mechanical analysis (DMA) test samples were printed using an ANYCUBIC DLP printer (Shenzhen, China) with 405 nm light source and power density of 509 (µW/cm^2^). Samples were printed with 100 s exposure time per layer and a layer thickness of 100 µm. Printed samples were post cured in a blue light oven (Form Cure, Formlabs Inc., Somerville, MA, USA) at 75 °C for 2 h, then thermally post-cured at 160 °C for 2 h. Samples with BHMF-M contents and neat IM were further post-cured at 200 °C for 1 h and 220 °C for 1 h. Figure 5 shows 3D printed bars for DMA testing, compact tension samples for fracture toughness testing, and an object with a more complex open shape that demonstrates these resin systems can be used to create parts with good resolution.

Extent of Cure. Functional groups of methacrylate and carbonyl were identified in samples before printing, after printing and after post-curing using a Nicolet 6700 FT-IR spectrometer in transmission (Thermo Fisher Scientific, Waltham, MA, USA). Mid-infrared spectra(M-IR) in the rage 650−4000 cm^−1^ were recorded with 32 scans at an 8 cm^−1^ resolution at room temperature using a deuterated triglycine sulfate (DTGS) detector. The conversion, α, was calculated by measuring the peak height (PH) of the methacrylate peak (943 cm^−1^) relative to that of the carbonyl peak (1717 cm^−1^) which remained the same during the reaction, following Equation (1) below. Photo-curing kinetics measurements, described in the results and discussion section, also use this equation.

(1) $$\alpha =1-\frac{{PH(t)}_{{943cm}^{-1}}}{{PH(t=0)}_{{943cm}^{-1}}}\frac{{PH(t=0)}_{{1717cm}^{-1}}}{{PH(t)}_{{1717cm}^{-1}}}$$

Table 2 summarizes experimental conversion measurements of the green parts and post-cured parts for each resin blend as characterized by mid-IR.

UV-Vis Spectroscopy: Spectrometric measurements of FM, IM and BHMF-M were obtained using an Ocean Optics (Peabody, MA, USA) USB2000 Spectrometer with a DH-2000-BAL Ocean Optics Deuterium-Tungsten Light Source in the range of 200–500 nm. Quartz cuvettes with 1 cm pathlength were used. The absorption spectra measurements were conducted at ambient temperature. The concentration of the resin solutions was 0.1%wt in acetonitrile. 

Dynamic Mechanical Analysis: DMA was performed on all polymer samples using a TA Instruments (New Castle, DE, USA) Model Q 800 instrument to evaluate the glass transition temperature (*T_g_*) and storage modulus (*E’*) at room temperature. DMA tests were conducted using single cantilever geometry at a frequency of 1 Hz and an amplitude of 10 μm. Sample dimensions were 3.05 mm × 13 mm × 17.5 mm. Poisson’s ratio was assumed to be 0.35 for all samples. The temperature was increased at ramp rate 2 °C/min from −120 °C to 240 °C. *T_g_* was obtained from both loss modulus and tan *δ* peak positions and when needed by analysis of the storage modulus as a function of temperature. Reported values of *T_g_* and *E’* are averages of two experiments, so standard deviations are not given for these data. 

Thermal Gravimetric Analysis (TGA): The thermal stability in an inert environment was investigated using a TA instruments (New Castle, DE, USA) Q50 TGA with a 10 °C/min ramp rate from room temperature to 800 °C in argon.

Fracture Toughness: The critical stress intensity factor (*K_Ic_*) and critical strain energy release rate (*G_Ic_*) values were obtained by testing seven compact tension specimens for each sample composition. A sharp pre-crack was made at the notch bottom using a fresh blade at room temperature before testing with a 0.5 mm/min crosshead speed at ambient temperature. *K_Ic_* and *G_Ic_* values were calculated following ASTM D 5045-99 [35].

## 3. Results and Discussion

### 3.1. Viscosity of IM-Based Resin Blends

In DLP printing, the fluidity of uncured resin is crucial as the resin must flow beneath the build platform as each layer is printed. Commercial DLP printers typically require resins with low viscosities, below 1000 cP, at the printing temperature [15]. The viscosities of IM-based resin blends at room temperature, as presented in Table 3, are all below 1000 cP, making them suitable for DLP printing. Additionally, it was observed that as the mass contents of the selected diluents increase, the viscosities of IM-based resin blends decrease.

### 3.2. Working Curves of IM-Based Resin Blends 

The depth of penetration (*D_p_*) is an important parameter in photocuring using a DLP printer. The *D_p_* values of different IM-based resin blends were determined using an ANYCUBIC DLP printer. The working curves of the resin blends were obtained by printing a single layer with varying exposure times (30 s to 200 s) using a 405 nm light source with a power density of 509 µW/cm², as measured by a radiometer (ILT2400, International Light Technologies, Peabody, MA, USA). The thickness of the printed layer was recorded for each resin blend at different exposure times, and plots of the curing depth (*C_d_*) as a function of the natural log of energy density (*E*) were generated. These curves were used to determine the critical energy (*E_c_*) and *D_p_* using Equation (2) [14].

(2) $$C_{d}=D_{p}\ln\left({E/E}_{c}\right)$$

The *D_p_* provides a measure of light attenuation during printing, while the critical energy is representative of the minimum energy required for the resin to reach gelation. The results are presented in Table 4, which lists the *D_p_*, *E_c_*, minimum printing time (Min ET) and the coefficient of determination (R^2^) associated with the curve fitting for each resin blend. It should be noted that significantly better fits, as measured by R^2^, were obtained for the BHMF-M blends compared to FM blends. This was particularly observed for blends with high FM content (20% and 40% IM), making the *D_p_*, *E_c_*, and Min ET values obtained for those compositions less reliable.

**Figure 6:**
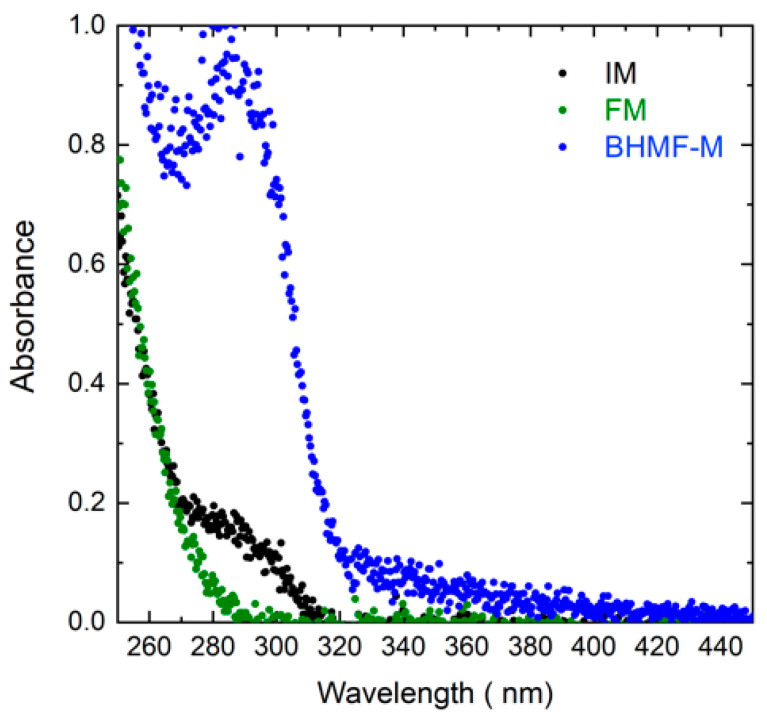
Absorbance spectra of BHMF-M, IM, and FM from 250 nm to 450 nm wavelengths.

The light absorbance of BHMF-M, IM, and FM resins was evaluated in the wavelength range of 200 nm to 500 nm as shown in Figure 6. The absorbance of the resin at the printing wavelength affects *D_p_*, which is crucial in controlling the cumulative dose profile. As shown in Table 4, the *D_p_* values of IM/FM blends are relatively high, generally greater than 400 μm, and relatively independent of composition. This is due to low absorbance for both IM and FM resin at the 405 nm wavelength. In contrast, the *D_p_* value for BHMF-M modified resin blends decreases monotonically as the BHMF-M content increases, with a value of only 80 μm for 100% BHMF-M resin. This is attributed to the higher absorbance of BHMF-M resin at 405 nm wavelength compared to that of IM, as shown in Figure 6. The *E_c_* and Min ET values also decrease with increasing BHMF-M content in the resin blends, suggesting a higher reactivity of BHMF-M compared to IM. Based on the analysis of the working curve parameters, a 100 s exposure time and a 100 μm layer thickness were selected as the printing parameters for all resin blends.

### 3.3. Thermo-Mechanical Properties of Green Parts

**Figure 7:**
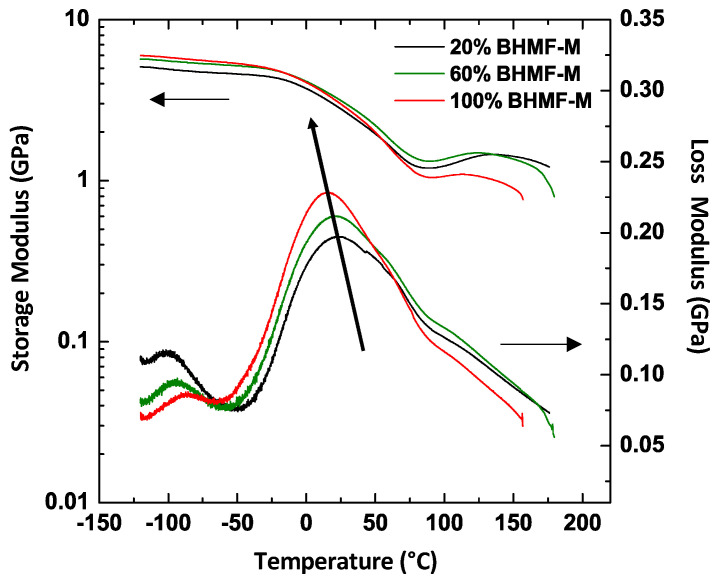
DMA plots for three BHMF-M/IM polymers showing that the *T_g_* of printed parts decreases as the weight content of BHMF-M increases.

The thermo-mechanical properties of the green parts were studied using DMA. The results indicated that all IM/FM resin blends resulted in green materials (printed part prior to post-curing) with *T_g_* close to room temperature, due to vitrification at the printing temperature. However, a clear trend was observed in the *T_g_* of BHMF-M/IM polymers as a function of composition. Figure 7 presents the storage and loss moduli of the printed green parts as a function of temperature for three BHMF-M/IM polymers (20% BHMF-M/80% IM, 60% BHMF-M/40% IM, and 100% BHMF-M/0% IM). The data indicate a decrease in *T_g_* as the content of BHMF-M increases. This behavior is attributed to the lower conversion of monomers in blends with a higher BHMF-M content as shown in Table 2, resulting from lower *D_p_* with increasing BHMF content.

**Figure 8:**
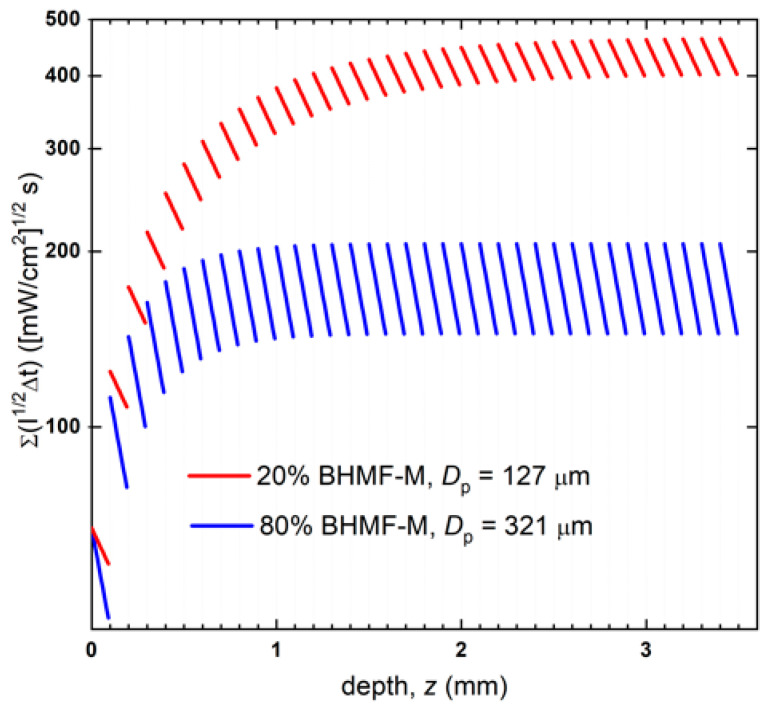
Cumulative applied reduced dose profiles of 20% BHMF-M/80% IM and 80% BHMF-M/20%IM resin blend printed bars.

Cure modeling of the printing process can be used to better understand differences in the behavior of resin formulation [36,37,38]. The differences in printed green material sample conversion were analyzed using our recently published model that combines calculated cumulative dose profiles and resin cure kinetics models to predict conversion [38]. The cumulative dose profile is controlled by the printing process and resin properties. Resin properties include *D_p_* and *E_c_*. The printing parameters include printer light intensity *I*_0_, layer exposure time Δ*t*, layer thickness Δ*z*, and number of layers *n*. In a DLP printing process, a series of layers is irradiated in sequence with well-defined characteristics: initially, the first layer (*L*1) is irradiated for a defined time *t*; then, the displacement of the moving stage allows light to irradiate the subsequent layer (*L*2). Note that because the light must exceed *E_c_* to cure the interface between *L*2 and *L*1, *L*1 inevitably is exposed to an additional light dose. The process continues until the object reaches the desired thickness. As each layer has a thickness equal to Δ*z*, the total applied dose *E_z_* along z of the printed object is the result of the sequential irradiation of the layers being built. The cumulative applied reduced dose *E’_z_* at depth *d* of the *i*^th^ layer can be calculated using Equation (3). Figure 8 shows the applied cumulative reduced dose profiles in 3.5 mm thick bars printed with the 80% BHMF-M and 20% BHMF-M modified IM resin systems.

(3) $$E_{z}^{’}=\sum_{k=i}^{n}{\left(I_{0}e^{-[d+(k-i)*∆z]/D_{p}}\right)}^{1/2}∆t$$

**Figure 9:**
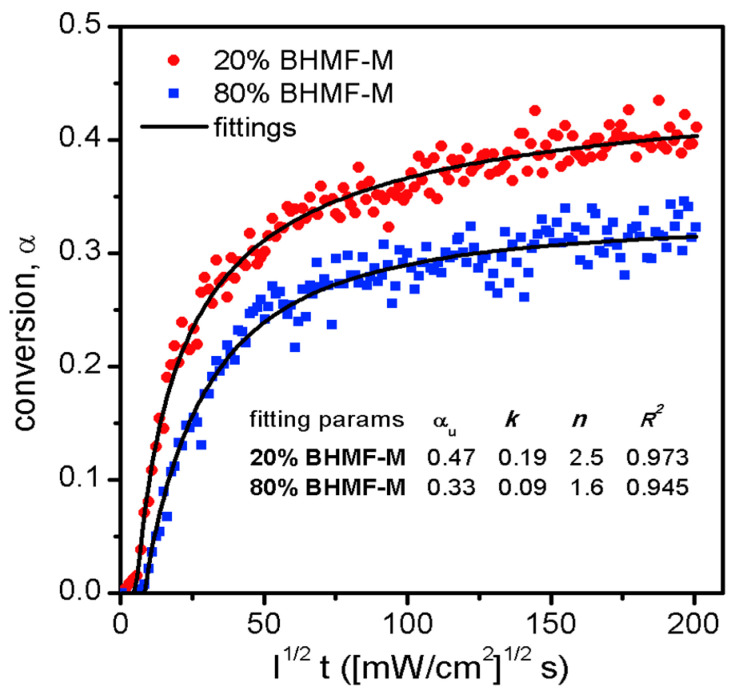
Cure kinetics of two resin blends, and the fittings to an *n*th order reaction kinetics model.

The cure kinetics of resin systems were investigated using photo-attenuated total reflectance Fourier transform infrared spectroscopy (ATR-FTIR). A single reflection diamond ATR unit (Golden Gate, Specac, Fort Washington, PA, USA) was integrated with a Nicolet 6700 FT-IR spectrometer (Thermo Fisher Scientific, Waltham, MA, USA) equipped with a temperature controller. A thin layer of the resin sample was irradiated with 405 nm light generated by an LED assembly, and its IR spectra were continuously collected by the FT-IR spectrometer. The light intensity that reached the bottom of the resin layer was pre-determined using a radiometer. The decrease in the methacrylate double bond peaks at 943 cm^−1^ was monitored as a measure of conversion, as described by Equation (1). The conversion profiles of the two resins were plotted against applied reduced dose and fitted to an n^th^ order reaction kinetics model, expressed by Equation (4). This kinetic model considers the ultimate conversion (*α_u_*), reaction rate constant (*k*), reaction order (*n*), and light intensity (*I*). The results are presented in Figure 9.

(4) $$\alpha =\alpha_{u}-{\left[\alpha_{u}^{\left(1-n\right)}-\left(1-n\right)kI^{\frac{1}{2}}t\right]}^{\frac{1}{1-n}}$$

**Figure 10:**
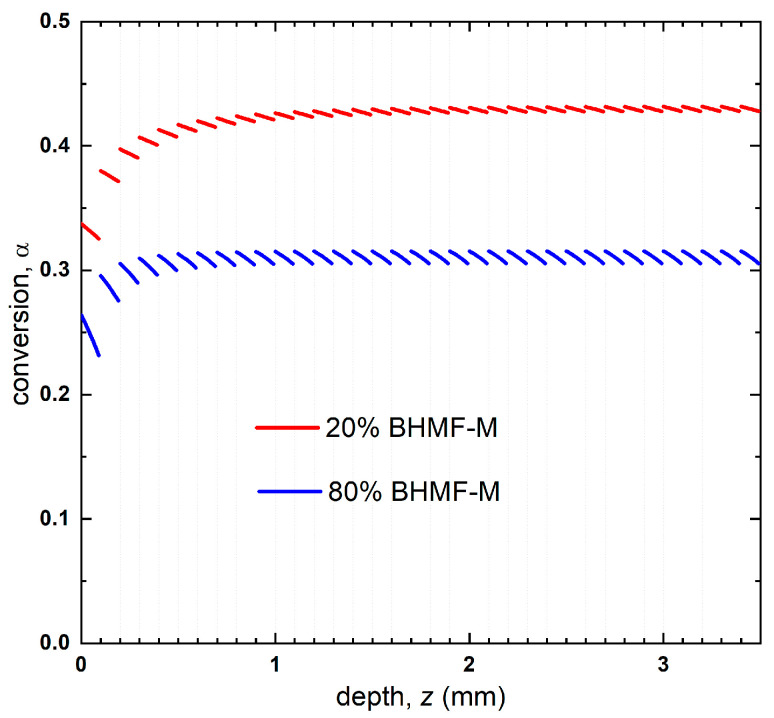
Predicted conversion profile of 20% BHMF-M/80% IM and 80% BHMF-M/20%IM resin blend printed bars.

The reaction kinetics, as represented by the conversion-dose relationship, allowed for the prediction of conversion profiles by mapping the conversion onto the reduced dose profiles, as depicted in Figure 10. The average through the thickness predicted conversion of the 20% BHMF-M polymer was found to be higher than that of the 80% BHMF-M polymer (0.42 compared to 0.31). The predicted conversions were in good agreement with the experimentally obtained through thickness conversion measurements for these two green parts (0.41 and 0.31 for 20% BHMF-M modified and 80% BHMF-M modified IM systems, respectively) as shown in Table 2. This difference can be attributed to the absorbance of the BHMF-M monomer at the printing wavelength. As the BHMF-M content increases, the light penetrates less through the part, resulting in lower conversion and hence a lower *T_g_* of the green parts. It is believed that the exotherm associated with the free radical polymerization reaction results in higher experimental degrees of conversions compared to predicted conversions for these systems.

### 3.4. Thermomechanical and Mechanical Properties of IM-Based Polymer

**Figure 11:**
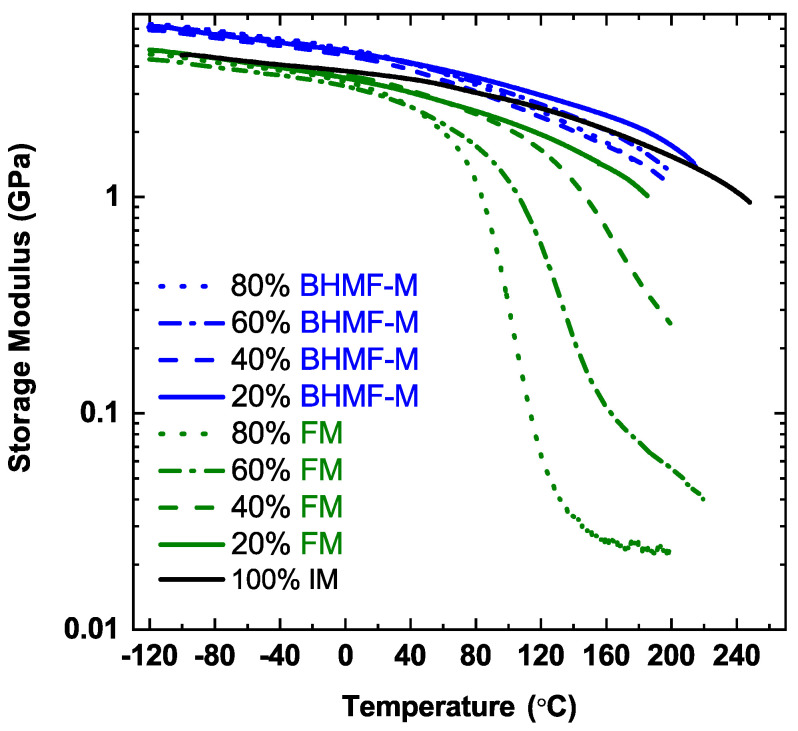
Storage modulus of FM/IM (green curves), BHMF-M/IM (blue curves) and neat IM (black curve) polymer networks.

The thermomechanical properties of post-cured resins were studied using DMA. Figure 11 presents the storage modulus as a function of temperature for IM-based systems, where the green curves represent FM/IM systems, the blue curves represent BHMF-M/IM systems, and the black curve represents the neat IM polymer. A summary of properties for these systems, including resin viscosity, *T_g_*, and *E’* at room temperature, can be found in Table 5. The *T_g_* values and estimates of *T_g_* reported in Table 5 were obtained using the storage modulus curves by taking the temperature corresponding to the inflection point when available or a 1 GPa cut-off value of *E’*. 

As shown in Figure 11, the neat IM polymer possesses a *T_g_* greater than 200 °C, characterized by the storage modulus curve which remains greater than 1 GPa even at 240 °C. The *T_g_* of IM-based polymer system decreases as the content of FM and BHMF-M co-monomers increases. As expected, the incorporation of mono-functional FM impacts *T_g_* more than BHMF-M, due to the more significant decrease in network crosslinking density resulting from the monofunctional monomer. The polymer that contains 20 wt.% of FM has a *T_g_* below 200 °C, whereas all the BHMF-M blends exhibit *T_g_* above 200 °C. 

IM-based resin blends result in polymers with high storage modulus at 25 °C, as shown in Table 5. The neat IM polymer system processes a storage modulus of 3.9 GPa at 25 °C. The FM-modified IM-based polymers show lower values compared to the neat IM system. The BHMF-M modified systems exhibit higher storage modulus compared to the neat IM polymer. This is due to the incorporation of the furan rings of the BHMF-M monomers into the backbone of the polymer networks. The result is consistent with findings in previous work on furan-based networks by our group and that of others that the furan moiety imparts high glassy modulus to thermosets [29,30,31,32]. A clear correlation between co-monomer content and polymer storage modulus was not evident for both FM/IM and BHMF-M/IM polymer networks.

**Figure 12:**
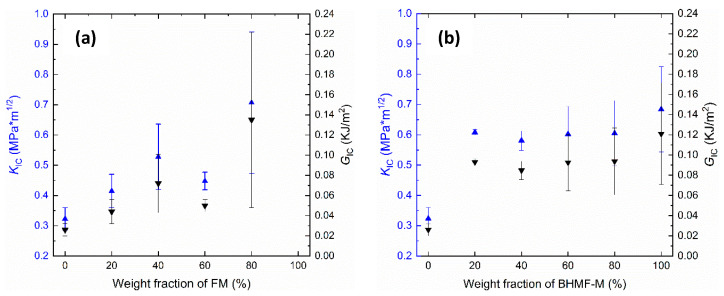
(**a**) *K_Ic_* and *G_Ic_* vs. weight fraction of FM in the FM/IM co-polymer systems; (**b**) *K_Ic_* and *G_Ic_* vs. weight fraction of BHMF-M in the BHMF-M/IM co-polymer systems.

The effects of co-polymerization on the fracture toughness of FM/IM and BHMF-M/IM polymers were studied by conducting fracture toughness tests. The critical stress intensity factor (*K_Ic_*) and critical strain energy release rate (*G_Ic_*) were calculated to determine the fracture properties of these polymer networks and were compared to those of the neat IM polymer. Results, as presented in Figure 12, show that the incorporation of FM and BHMF-M into IM provides a significant increase in fracture toughness. It was found that the BHMF-M imparted a greater enhancement in both *G_Ic_* and *K_Ic_* values compared to the FM co-polymers. With regards to the FM/IM systems, both *G_Ic_* and *K_Ic_* values increased as the content of FM increased, except for the 60% modified system which exhibited lower values. The addition of the BHMF-M co-monomer at concentrations as low as 20% resulted in a significant increase in *G_Ic_* fracture toughness to values at or greater than 100 J/m^2^. A significant increase was not observed with further increases in BHMF-M concentration. 

**Figure 13:**
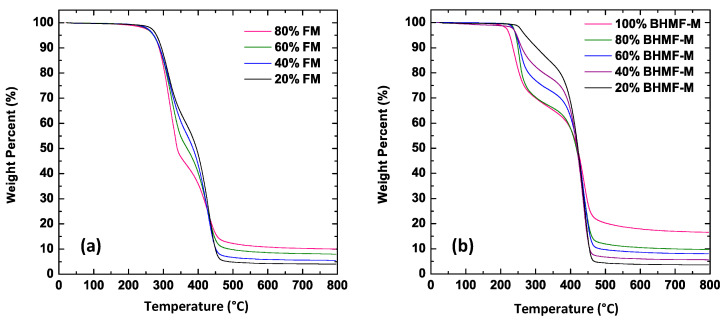
(**a**) TGA thermograms of cured samples of FM/IM polymer systems; (**b**) TGA thermograms of cured samples of BHMF-M/IM polymer systems.

The thermal degradation behavior of FM/IM and BHMF-M/IM polymer systems was investigated using TGA in an argon atmosphere. The TGA thermograms of the cured FM/IM and BHMF-M/IM samples are shown in Figure 13a,b, respectively. The thermal decomposition temperatures corresponding to a 5% weight loss (*T_d5_*_%_) decreased as the weight percent of BHMF-M increased, ranging from 272 °C for 20% BHMF-M to 225 °C for neat BHMF-M polymer. This trend suggests that the furan moiety is less thermally stable than the isosorbide building block. The *T_d5_*_%_ values for single component crosslinked polymers are significantly higher than the *T_g_* of those systems −85 °C higher for IM and 75 °C higher for BHMF-M. However, the *T_d5%_* values are much closer to the *T_g_* of the blends, suggesting that those compositions’ decomposition would affect glassy state performance at temperatures close to *T_g_*. As for the FM/IM polymer systems, the thermal decomposition temperatures of the FM-modified networks were lower than those of the neat IM system. However, increasing the FM content did not result in a lower thermal decomposition temperature. In comparison, the FM-modified polymer systems exhibited better thermal stability than the BHMF-M-modified systems, as evidenced by a higher thermal decomposition temperature and a slightly lower degradation rate. In all cases, *T_d5%_* was found to be much higher than *T_g_*. These observations suggest that the furan moiety has a greater impact on the thermal degradation behavior of the polymer when crosslinked into the backbone structure. The char yields of both FM and BHMF-M modified polymer systems showed that the furan moiety imparts a higher char yield than the isosorbide building blocks. The BHMF-M modified networks had a slightly higher char yield possibly due to a higher crosslinking density compared to the FM-modified networks.

## 4. Conclusions 

In this study, fully bio-derived isosorbide-based resin formulations were examined as a replacement for BPA-derived methacrylates and styrene copolymers in 3D printing. The viscosity of the resin blends was low (below 0.12 Pa·s at 20 °C). The working curves and curing kinetics were analyzed to determine optimized printing parameters. The results showed that BHMF-M acts as a photo absorber at the printing wavelength, leading to reduced overall conversions compared to FM-modified IM resins in as-printed parts. After post-cure, however, the differences in conversion became negligible. The addition of FM and BHMF-M co-monomers at low concentrations increased the fracture toughness of IM to levels suitable for practical applications (*G_IC_* > 100 J/m^2^). In terms of thermomechanical properties, BHMF-M/IM co-polymers had *T_g_* values greater than 200 °C (determined by the *E’* curves) with good thermal stability, whereas the incorporation of mono-functional FM reduced *T_g_* significantly (*T_g_* below 200 °C for 20 wt.% FM). Overall, IM-based resin systems show great potential for use in bio-based polymers for additive manufacturing with advantageous properties. 

## Figures and Tables

**Table 1:** Monomer viscosity, polymer storage modulus *E*’, *T_g_* identified as the maximum of loss modulus (*E*”) and of tan *δ*, for isosorbide methacrylate (IM), furfuryl methacrylate (FM) and 2,5-bis(hydroxy-methyl) furan methacrylate (BHMF-M).

	Viscosity @ 21 °C (cP)	*E*′ @ 25 °C (GPa)	*T_g_*, *E”* (°C)	*T_g_*, tan *δ* (°C)
IM	157 [20]	~4 [20]	-	>245 + 9 [20]
FM	4	2.9 ± 0.6	98 ± 4	124 ± 6
BHMF-M (with wt. 20% Styrene)	16	5.2	-	150

**Table 2:** Extent of cure of BHMF-M/IM and FM/IM resin blends.

Mass % IM	Mass % FM	Mass % BHMF-M	Green Part Conversion %	Post-Cure Conversion %
100	0	0	-	85
80	20	0	69	85
60	40	0	71	84
40	60	0	70	85
20	80	0	43	85
80	0	20	41	86
60	0	40	44	86
40	0	60	34	85
20	0	80	31	80
0	0	100	38	84

**Table 3:** Compositions and viscosity of IM-based resin blends with 0.7 wt% of PPO.

Mass % IM	Mass % FM	Mass % BHMF-M	Viscosity (cP)
100	0	0	157 [20]
80	20	0	32 + 4
60	40	0	7 + 0
40	60	0	7 + 2
20	80	0	5 + 1
80	0	20	107 + 10
60	0	40	92 + 5
40	0	60	89 + 35
20	0	80	69+ 5
0	0	100	61 + 6

**Table 4:** Depth of penetration (*D_p_*), critical energy (*E_c_*) and minimum printing time (Min ET) of IM-based resin blends.

	FM Blends					BHMF-M Blends					
IM content (%)	20	40	60	80	100	0	20	40	60	80	100
*D_p_* (μm)	413	725	513	498	503	80	127	159	196	321	503
*E_c_* (mJ/cm^2^)	9.5	16.5	9.7	9.2	8.4	6.0	8.7	9.1	7.9	9.6	8.4
Min ET (s)	18.7	32.5	19.0	18.0	16.5	11.7	17.1	17.9	15.4	18.9	16.5
R^2^	0.691	0.916	0.920	0.954	0.986	0.945	0.980	0.991	0.977	0.995	0.98

**Table 5:** Viscosity, *T_g_*, and *E*’*_RT_* f FM/IM and BHMF-M/IM blends.

	Wt (%)	Viscosity (21 °C) (cP)	*T_g_*, *E’* (°C)	*E’_RT_* (GPa)	*T_d_*_5%_ (°C)
IM	100	157 [20]	~220 [20]	3.9	305
BHMF-M	20	107	>200	4.5	272
	40	92	>200	4.4	243
	60	89	>200	4.2	245
	80	69	>200	4.5	246
	100	61	150	3.6	225
FM	20	32	200	3.3	280
	40	7	170	3.5	272
	60	7	140	3.0	272
	80	5	110	3.1	273

## Data Availability

The research data are available upon request.
